# Myopia and daylight—A combination of factors

**DOI:** 10.3389/fmed.2025.1481209

**Published:** 2025-07-02

**Authors:** Richard Hobday, Mariëlle Aarts, Christian Cajochen, Lenka Maierova, Mirjam Münch, Werner Osterhaus, Oliver Stefani, Katharina Wulff

**Affiliations:** ^1^Hobday Research, Cwmbran, United Kingdom; ^2^Building Lighting Group, Department of the Built Environment, Eindhoven University of Technology, Eindhoven, Netherlands; ^3^Centre for Chronobiology, Psychiatric Hospital of the University of Basel, Basel, Switzerland; ^4^Research Cluster Molecular and Cognitive Neurosciences, University of Basel, Basel, Switzerland; ^5^Czech Technical University in Prague, University Centre for Energy Efficient Buildings, Buštěhrad, Czechia; ^6^Strategic Real Estate Development, Building Division, Vicariate General of the Catholic Arch- diocese of Paderborn, Paderborn, Germany; ^7^Lucerne School of Engineering and Architecture, University of Applied Sciences and Arts, Lucerne, Switzerland; ^8^Department of Molecular Biology, Umeå University, Umeå, Sweden; ^9^Wallenberg Centre for Molecular Medicine (WCMM), Umeå University, Umeå, Sweden

**Keywords:** pseudomyopia, near work, anxiety, posture, daylight, intensive education

## Abstract

The incidence of myopia among school children has risen markedly over the last three decades. In urban areas of South and East Asia, as many as 80–90% of young adults are now myopic. This trend is occurring elsewhere around the world. During the COVID-19 lockdowns, children in many countries were confined indoors and spent an undue amount of time exposed to television screens, computers, and mobile devices. This resulted in an acceleration in the incidence and progression of the condition. Myopia is a significant public health issue as it is a leading cause of blindness and other vision problems. Yet the underlying mechanisms that produce the condition remain elusive. Pseudomyopia has recently been proposed as an independent risk factor for myopia. We hypothesize that pseudomyopia induced by prolonged close work, stress, and anxiety combines and is further amplified by chronically low ambient light levels. If time spent outdoors in daylight is restricted, the effects worsen and together may play a significant part in myopia epidemics.

## 1 Introduction

Myopia results from abnormal axial lengthening and other changes in the eye, causing light to focus in front of the retina rather than directly onto it, which leads to blurred distance vision. Notably, 2.5 billion people are living with myopia globally, and this figure is set to rise to 4.75 billion by 2050 (1). Increasingly, people with moderate myopia are developing high myopia. In such cases, elongation of the eye increases the risk of permanent visual loss through degenerative changes in the eye. Even mild to moderate myopia significantly increases the risk of such complications (2). If unaddressed, the global myopia epidemic will harm the physical (3) and psychological wellbeing (4), and the economic prospects (5) of much of the world's population.

Genetic and environmental factors are involved in myopia, although genetic change does not account for the rapid increases in prevalence during epidemics (6). Intensive education and limited time spent outdoors in daylight are generally recognized as the most critical contributors (7). Evidence for other risk factors investigated is weaker (8), and it is unclear how the mechanisms that may be involved combine to disrupt the normal growth of the eye (8). Spending time outdoors during the day has a protective effect for reasons that are not fully understood (9). The most significant factor appears to be that light outdoors is brighter than indoors and has a broader spectrum (8). Optical differences between indoors and outdoors may also play a part. Reduced accommodation due to more distance viewing outdoors, the pattern of retinal defocus generated outdoors, and a higher spatial frequency may account for some of it (2, 10).

The protective effect of bright light is widely attributed to the neurotransmitter dopamine. The dopamine hypothesis proposes that retinal dopamine release, stimulated by daylight exposure, inhibits axial elongation (11, 12). Blue light is being tested as a potential stimulant for retinal dopamine production (13) and has demonstrated a myopia-inhibiting effect (14). Violet light has done the same to a modest level (15, 16). Also, rather than specific wavelengths, some researchers are investigating the effectiveness of exposure to the entire visible spectrum in myopia treatment (17, 18). Meanwhile, red-light therapy has proven its effectiveness as a short-term treatment for myopia progression (19, 20), but there may be safety issues (21). Also, the long-term effects and safety of pharmacological interventions for myopia progression, such as atropine eye drops, are not clear. Nor are they for some of the contact and spectacle lenses designed to prevent progression (22). Another unresolved issue is whether daylight can slow down or stop myopia from progressing (23–25).

**Figure 1:**
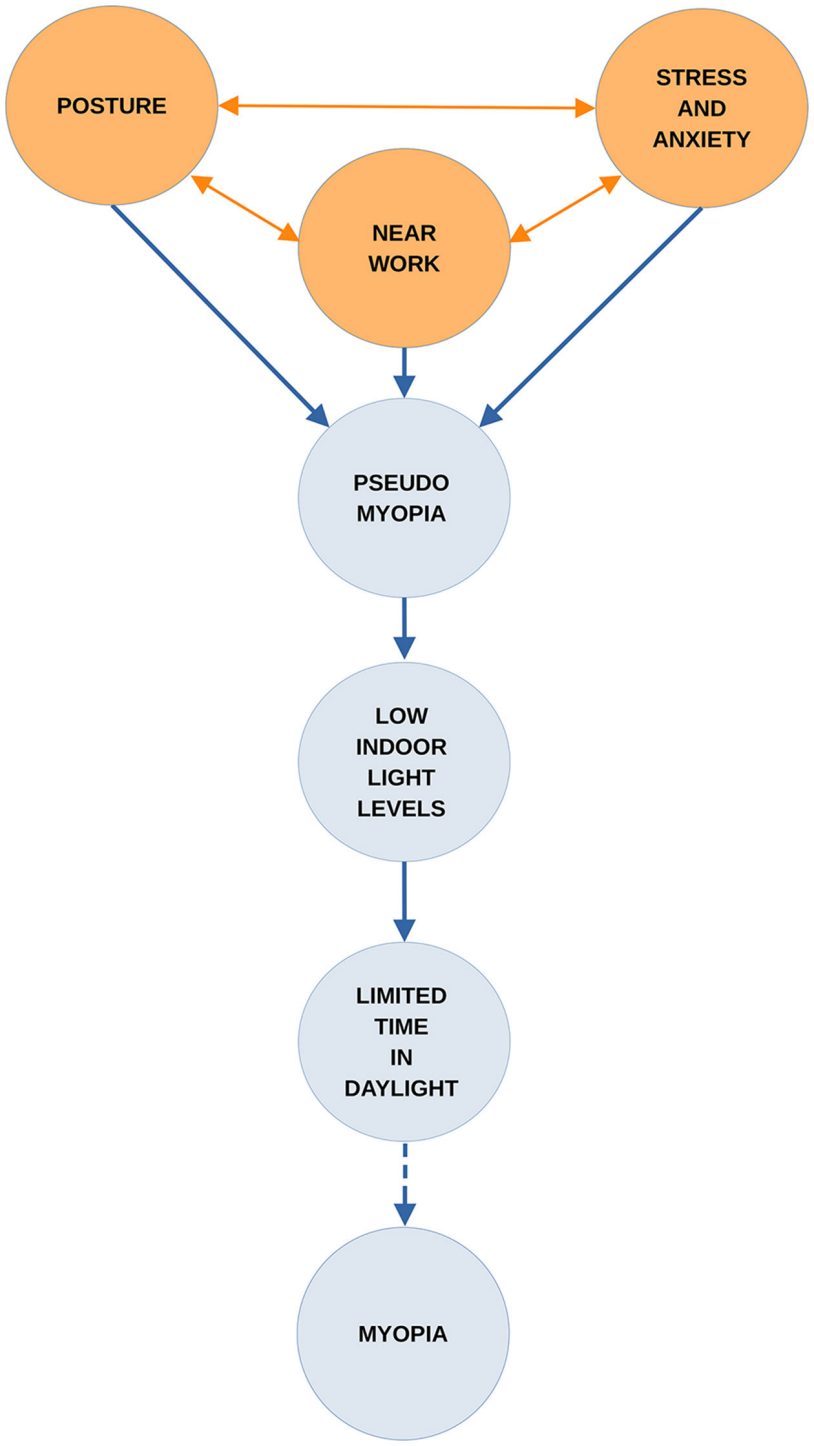
The combined effects of suggested factors leading to myopia.

Until the underlying mechanisms are better understood, it will remain a challenge to develop fully effective strategies for myopia control (26). This paper begins by reviewing the background to an earlier hypothesis. This informs the one presented here which is that when both near work and emotional symptoms are present the pseudomyopia induced by them can combine and increase in strength. Furthermore, the effects may be amplified if this union occurs under low light levels. If children's access to daylight outdoors is also limited, the confluence of these risk factors could play a significant part in myopia onset and progression. The paper then examines the roles of pertinent risk factors in the onset and progression of myopia. A visualization of how these may interact and amplify the development of myopia is given in Figure 1.

## 2 Background

Over the last three decades, the prevalence of myopia in urban areas of South and East Asia has increased to the point where 80–90% of children leave school with the condition (27). Other regions of the world are following this trend. When populations move from rural to urban areas, and when the duration and competitiveness of their children's education increases, so does myopia (27). Urbanization is considered to be a potential risk factor (7, 8). Cohn first identified this in the 1860s. The evidence came from the findings of a landmark survey of the eyesight of over 10,000 Prussian school children (28).

Cohn found that children attending schools in towns had more myopia than children in comparable schools in rural areas. He also discovered that the longer children attended school the more likely they were to become myopic. Also, as the educational pressures placed on them increased, myopia became more common and more severe (29). In Prussia's academic High Schools, or Gymnasiums, the percentage of myopia went up steadily from the first year of attendance to the sixth. More than 50% of the children examined in their final year at these schools were myopic (29). Cohn also carried out the first investigation of the effects of classroom lighting on myopia by comparing the amount of daylight in classrooms with the number of myopic children studying in them. He concluded that schools with small windows, or overshadowed by adjacent buildings, had the highest rates. Those situated in the narrowest, darkest streets were the worst affected. Cohn also concluded, as others subsequently did (29), that reading and writing in dimly lit environment was one of the most important factors for myopia onset. Another was leaning forward while doing so. According to Cohn, an upright posture had a protective effect (29).

Cohn recommended good lighting in classrooms and exercise and recreation outdoors in daylight. Education departments arranged their schools for this for many years afterwards (30). In Britain, surveys conducted in London schools during the early 1900s found that girls were more susceptible to myopia than boys (31). This was attributed to the close work girls did in schools, especially needlework, and time spent indoors at home helping with domestic chores. Parents allowed boys to play outdoors in the city's streets but were reluctant to let girls do so (31). This difference in myopia prevalence between the sexes is still found in urban centers in some countries (32).

Other distinguished ophthalmologists, notably Fuchs and then Duke–Elder considered excessive near work to be the most crucial factor in the etiology of myopia (33, 34). They also appreciated the importance of proper illumination and exercise outdoors in daylight in preventing the condition. Fuchs and Duke–Elder also shared Cohn's concerns about the harm done to children by the excessive amount of study many had to undertake, both at school and afterwards at home. However, they were unsure about the mechanisms that caused myopia (33, 34). Cohn had argued that spasm of accommodation was the precursor of chronic myopia (29), but his theory was not accepted; nor does it appear to have been tested during his lifetime. In addition, neither Fuchs, Duke–Elder, nor Cohn directly addressed the influence of academic stress and anxiety on children's eyesight. This came later, in the 1940s, with the introduction of psychosomatic medicine to ophthalmology (35).

Cohn recognized that heredity played a part in the myopia that some children developed. However, in his judgement, this was not the underlying cause in most cases. Heredity did become the prevailing orthodoxy in the 1960s, following the publication of an influential study which asserted that myopia was almost entirely genetic in origin. This was taught to students for many years (36). One consequence was that designing schools for daylight was no longer a priority. Another was that the protective effect of daylight received little research attention (30).

During the COVID-19 pandemic, many countries used nationwide lockdowns as a quarantine measure. Children were confined in their homes and educated online. Exposure time to digital screens for virtual learning and recreation increased while outdoor activities were restricted. There are reports of an acceleration in myopia progression and incidence in children following the pandemic (37–40) and also in the psychological stress they experienced (41, 42). A more positive finding was the identification of pseudomyopia as an independent risk factor for myopia (43). Pseudomyopia, which is also known as transient myopia, ciliary spasm or, as Cohn referred to it, accommodation spasm can follow prolonged near work (44). It can also be caused by head injury and psychological stress, notably fear and anxiety (44, 45). If confirmed as a risk factor in future research, this would explain some of the rapid onset of myopia and its progression during pandemics. It would also assist in understanding how the etiology of myopia relates to daylight exposure, or lack of it.

## 3 Near work and myopia

Near work has long been regarded as a major risk factor for myopia. Johannes Kepler noted this in students 400 years ago (46). Others have argued the opposite: that near work has no influence on myopia (47) or that there is not enough evidence to prove it (48). Currently, many studies support the connection, but the literature is contradictory (49). For example, in 2021, the results of a study on primary students in Wenzhou, China, found that high levels of outdoor exposure had a marked influence by lowering the risk of myopia onset. Near work had none (50). However, the findings of a pilot study of the effects of learning to read and of sustained intensive near work at a very early age suggest the opposite. They may be strong enough to override the protective effects of time spent outdoors (51). Clinical trials are scarce because participants' adherence to study protocols and the monitoring of this is problematic. Nevertheless, the authors of a recent systematic review and meta-analysis of near work and myopia have concluded there is a statistically significant association, both in infants and adults (52). Meanwhile, some countries with high rates of myopia in children have measures in place to control near work (53).

## 4 Near-work-induced transient myopia

Near-work-induced transient myopia (NITM) is a common form of pseudomyopia. It is a short-term myopic shift in distance vision that occurs straight after prolonged near work (54). The shift, or delay in accommodation relaxation, can take place after a few minutes or periods of several hours. The interruption of near tasks can prevent it. People with myopia are more susceptible to NITM than people with normal vision. Also, those with progressing myopia have greater NITM with a longer decay time than people with stable myopia (55). In 2008, pseudomyopia, in the form of NITM, was identified as a possible component of myopia (56). It was suggested that there may be an additive process at work, with residual NITM contributing to the transition to permanent myopia (57). Clinical trials were proposed to investigate whether NITM is involved in the genesis of permanent myopia (57). Such trials do not appear to have been undertaken. One explanation is that there is no experimental proof that accommodation interacts with the emmetropisation process (58). However, it now seems that temporary myopia can become permanent. The study that discovered this began in Shandong province, China, in September 2020. It recruited a cohort of non-myopic children. A 6-month follow-up found that 21.1% of children with pseudomyopic eyes developed myopia. Of non-myopic and non-pseudomyopic, only 3.8% had developed myopia (43). The authors of this study identified pseudomyopia as an independent risk factor for the condition for the first time. But they also noted there is no evidence of a direct path from transient to permanent myopia (43).

## 5 Low illuminance and myopia

For many years, research into the effects of near work under low illuminance levels received limited attention and the idea that reading in dim light damages eyesight became unfashionable (59). Several studies have since been undertaken to determine how the human eye develops under low levels of light exposure. In 2012, one finding from the Beijing Childhood Eye Study was that low illumination during reading was associated with a higher prevalence and amount of myopia (60). Findings from The Sydney Adolescent Vascular and Eye Study (61) show that 6-year-old children with little exposure to daylight have a 5.2 times greater risk of developing myopia. Significantly, this could rise to as much as 15.9 times if they also perform close-up work. The control group for this longitudinal study, which had a 5-year follow-up, consisted of children who spent significant amounts of time outdoors and little time on near-vision activities. In 2015, a longitudinal study of myopic and non-myopic Australian children grouped them by their daytime light exposure, which was split into three exposure levels: low, medium, and high. There was significantly faster axial eye growth over time among children who experienced the lowest average daytime light exposure, below an average of 459 lux, compared to children who experienced higher levels (62).

Several studies support the theory of an association between light levels typically found indoors and myopia (63–66). Further evidence comes from reports of seasonal differences in myopia progression, with slower progression in summer (67–70). The results of a randomized control trial from 2015 in which the ambient lighting of refurbished classrooms was increased to 558 lux at the desk and 440 lux at the blackboard showed a marked effect. It reduced the prevalence of new-onset myopia in the intervention group to 4% compared to the control group's onset rate of 10%. Decreases in axial growth and refraction were also reported (71).

## 6 Digital eyestrain, posture, and myopia

Children using digital media during COVID-19 lockdowns may have been susceptible to both myopia and digital eye strain (DES) (72, 73). This is also variously called computer vision syndrome (CVS), visual fatigue (VF), and eye strain, and is part of asthenopia (74). The ocular symptoms of DES include blurred vision from accommodative strain, dry eyes, red eyes, altered blinking characteristics, eye pain, and headache (75–77). Musculoskeletal disorders, such as neck and shoulder pain, are also among the symptoms of DES. These are caused by postural problems, poor ergonomics, and work practices (74). An inadequate posture may also cause myopia, as Cohn argued in his book *Hygiene of the Eye in Schools*. This states that the adverse effects of a bad posture in children are spinal curvature and shortsight. The latter results from continuous stooping of the head when looking at near objects (29).

Postural changes that activate the neck muscles are known to affect the eye. One study has found that reading with the head tilted forward and the neck at an angle of 45° increases both intraocular pressure and NITM compared to reading with the head held upright (78). Additionally, there is evidence that people with existing neck and shoulder symptoms are more susceptible to eye discomfort when performing near work. This is related to the accommodative demands on their eyes (79). Adopting an upright posture when performing near work reduces the risk of fatiguing the upper trapezius muscle in the neck, which can otherwise lead to neck and shoulder pain (80). However, if there is eye strain, and the eye's ciliary muscle is forced during prolonged near work, this can activate trapezius muscle activity and fatigue it (81, 82). To compound the problem, low-level and acute stress can trigger the trapezius muscle (83, 84). Fortunately, research suggests that in addition to NITM, an upright posture may improve resilience to stress and reduce depression and anxiety (85, 86).

## 7 Anxiety, psychological stress, and myopia

Depression and psychological stress are associated with several ophthalmic conditions, such as dry eye disease and glaucoma (87, 88). The most common mental health illness associated with pseudomyopia is generalized anxiety disorder (GAD) (89). Pseudomyopia can also manifest following both chronic and acute stress. During World War II, a Medical Officer in the United States Navy reported that ciliary spasms had significantly increased during wartime. This was due to emotional trauma and cost the United States Army and Navy many thousands of man-hours (90). In 1959, the author of a review of 21 cases of pseudomyopia concluded they were brought on by sheer fright. Or, taking on a challenging task in which failure would entail a loss of face (45). Pseudomyopia is also reported to have developed among 30% of residents whose sight was tested following an earthquake in Armenia in 1988 (87, 91).

Increased anxiety levels are common among adolescents and students with myopia. As the degree of myopia increases, so do the anxiety levels (92, 93). Anxiety and stress are also associated with the prolonged use of digital screens (94). A Canadian study has found that the increase in digital media use and TV viewing that occurred during their lockdown was linked to symptoms of anxiety and depression in children and adolescents (95). Prior to COVID-19, anxiety was already common among children and especially those in highly competitive learning environments (96). This is not a new phenomenon. In the 1940s, pseudomyopia was referred to as a relatively common ophthalmic condition, especially among school children, and there was an interaction between near work, stress, and anxiety:

“*A typical example of ciliary spasm is seen in the young student who is on the verge of failure in school. Because of his poor scholastic ability he is forced to spend more than the average amount of time in study. His anxiety over his incipient failure causes him to work under more and more pressure until a vicious circle of more reading and greater anxiety results in a spastic myopia of greater or less degree*” (97).

## 8 Educational styles and children's eyesight

A Japanese study published in 2021 suggests that when the academic burden on school children is reduced, it prevents eye damage. Japan has a highly competitive education system. A culture of *exam hell* is reported to have been problematic in Japanese schools, and in 2002, a less intensive school curriculum was introduced in Japan to try to address this (98).

The so-called Yutori educational policy reformed Japan's first 9 years of compulsory education. It created a more relaxed learning environment, which dispensed with Saturday classes and provided more opportunities for outdoor play (98). The Yutori policy remained in place until 2012, when a more intensive academic system was reintroduced. A retrospective observational study found that myopia progression and an increased prevalence of high myopia occurred only when high-pressure education was in place and not under the Yutori system (98).

Some countries, such as Australia, have maintained high academic standards with lower rates of myopia among school children than those reported elsewhere (99). In Australia, this has been attributed to the lifestyle and educational system (100). Another notable example is Norway, where a study of 16–19-year-old Norwegians found 13% of them were affected by myopia (101). Why myopia is less common in Norway than in many other countries is unclear at present. One factor is that in Norway, young children are outdoors for long periods. According to a survey of Norwegian kindergartens, children spend more than two-thirds of their time outside during the summer and about a third of the winter semester. Norway's kindergartens are designed for outdoor play, and no targets are set for children as they progress toward readiness for school (102). Preparation for academic learning is limited (103).

## 9 Discussion

The epidemic of myopia that occurred during COVID-19 is not without precedent. A notable outbreak occurred in Canada during the 1960s when Inuit children were taken from their families and forced to attend boarding schools. There followed an epidemic of myopia, which at the time was believed to be genetic in origin. Research was later published which, proposed the school environment as a significant factor in this epidemic and not genetics (104).

A recent paper has revisited the myopia epidemic, and the authors proposed that the removal of First Nation children from a traditional, open-air way of life to one of enforced near work indoors, under bad lighting, and a lack of time outdoors in daylight contributed to the sudden increase in myopia (105). Another component of the epidemic may have been the extreme psychological and physical distress these children experienced in residential schools (105).

It is tempting to speculate that this epidemic occurred because many of the factors proposed in this paper were present and may have combined to produce it. But it is not evident how in more favorable circumstances daylight outdoors would have made the transient myopia in these children disappear and emmetropia return. To do so it would have to achieve two things simultaneously: alleviate the adverse effects of near work on accommodation as well as those from stress and anxiety.

Some of the protective effects of daylight on vision could have a psychological basis. Daylight can improve mental health (106). A lack of it can disrupt the body's circadian rhythms and lead to sleep disorders, depression, and anxiety. If this disruption occurs, it could also trigger circadian dysregulation in the eye, which is a potential risk factor for myopia (107). In addition, the dopamine that entrains intrinsic retinal circadian rhythms can also affect mood, suggesting an interaction (108). If there is one, this would further support the hypothesis that dopamine is central to daylight's protective effect.

Another possible explanation is that just being outdoors—away from the classroom, playing with friends, viewing distant objects, and maintaining an upright posture—may be sufficient to reduce stress and anxiety levels. This hypothesis would not be difficult to assess. However, the part played by postural alignment in myopia may be. There has been little research on the different effects of slumped and upright postures on emotions (86). Equally, it has been recognized for some time that the relationship between the musculoskeletal system and that of accommodation may be bidirectional (109). Other pathways that lead to myopia may share this characteristic. There may also be interactions between them, which could become disrupted should the demands placed upon them become excessive. Daylight's protective effect may be to instigate processes that prevent these interactions.

While that requires further investigation, research is beginning to identify what occurs when several risk factors for myopia are present and access to daylight is limited. Following COVID-19 lockdowns in China, the cumulative effects of some protective measures were found to have been more significant than those from each one taken individually (110). The findings suggest that having good illumination and an upright posture when reading, resting the eyes, adequate sleep, spending time outdoors, regular exercise, and a nutritious diet can significantly reduce myopia progression and incidence in children. This highlights the importance of examining risk factors in combination rather than in isolation.

Some of the most pressing research questions concern the education of children and students. Finding a way of teaching them both at school and at home without damaging their vision must be a priority. There may be a technological solution to this that waits to be discovered. In the meantime, parents and teachers should be made aware of the measures that can protect children's vision and the importance of doing so. In some cultures, there is a belief that allowing children more time outdoors will adversely affect their education (53). It is noteworthy that the Norwegian education system places a strong emphasis on children's overall wellbeing (103) and, although not designed to do so, protects young children from myopia, which others do not. This should be investigated. Outdoor learning and the oral tradition of teaching may have much to commend them where eyesight is concerned (111). It would also be helpful if some of the terms used in myopia research, such as DES and near work, were more clearly defined.

The hypothesis presented in this paper sets out a pathway through which several mechanisms may combine and increase the risk of myopia onset and progression, via pseudomyopia. There is now some support for key elements of it, such as the transition to myopia from pseudomyopia and the additive nature of risk factors involved. While testing the hypothesis presented here should be possible, identifying which specific aspects of outdoor daylight exposure prevent the onset and progression from pseudomyopia to myopia would be more challenging, particularly because these factors may be interacting simultaneously.

A significant limitation of this study is that, to date, there is not enough scientific evidence in the literature to perform a systematic review and a meta-analysis, which would support or refute our hypothesis. Some of our conclusions are drawn from the observations of historical figures who were working at different times, following different protocols. In several cases, the research on which their observations and recommendations were based was never validated. However, there would appear to be sufficient evidence to suggest this hypothesis merits further investigation.

## Data Availability

The original contributions presented in the study are included in the article/supplementary material, further inquiries can be directed to the corresponding author.
